# RNAi Screening-based Identification of USP10 as a Novel Regulator of Paraptosis

**DOI:** 10.1038/s41598-019-40982-z

**Published:** 2019-03-20

**Authors:** Jin Yeop Kim, Dong Min Lee, Hyun Goo Woo, Ki Deok Kim, Hong Jae Lee, Yong-Jun Kwon, Kyeong Sook Choi

**Affiliations:** 1 Department of Biomedical Sciences, Department of Biochemistry and Molecular biology, Ajou University Graduate School of Medicine, 16499, Suwon, Korea; 2 Institut Pasteur Korea, Seongnam, Korea; 3 Department of Physiology, Department of Biomedical Sciences, Ajou University School of Medicine, Suwon, Korea; 4 Early Discovery, Ksilink, 16, rue d’Ankara, 67000 Strasbourg, France

## Abstract

Accumulating reports demonstrate that apoptosis does not explain all the effects of cancer therapy due to the innate and acquired apoptotic resistance of malignant cancer cells. Recently, paraptosis, a type of programmed cell death accompanied by dilation of mitochondria and/or the endoplasmic reticulum (ER), has garnered interest in cancer research as an alternative way to kill apoptosis-resistant cancers. We describe here the adaptation and validation of a high-content cell-based assay to screen and identify novel paraptotic regulators employing the malignant breast cancer cells undergoing curcumin-induced paraptosis. We used YFP-Mito cells, which express fluorescence selectively in mitochondria, to select paraptosis-related genes whose corresponding siRNAs appeared to modulate mitochondrial dilation, a morphological feature of paraptosis. From the selected 38 candidate genes, we chose ubiquitin specific peptidase 10 (USP10), a ubiquitin specific protease, as a strongly active candidate that warranted further evaluation of its involvement in paraptosis. We found that both siRNA-mediated knockdown of USP10 and treatment with the USP10 inhibitor, spautin-1, effectively attenuated curcumin-induced paraptosis. This systematic assay, in which a siRNA library is screened for the ability to ameliorate paraptotic changes in mitochondria, may enable researchers to identify potent regulators of paraptosis and new candidate genes/drugs to combat malignant breast cancer.

## Introduction

Breast cancer is one of the most common cancer types that cause oncologic morbidity and mortality among women worldwide^1^. Currently, breast cancers are treated with tailored combinations of surgery, chemotherapy and radiation^2^. Although ongoing research is seeking to develop more effective therapeutic strategies with minimal side effects, we lack a specific targeted agent for the treatment of triple negative breast cancer (TNBC), and the treatment options for TNBC patients are limited^3^. To identify novel targets that will enable us to effectively kill TNBC cells, we need to develop scalable strategies with powerful tools. Since malignant cancer cells, including TNBC cells, are resistant to pro-apoptotic treatments, it could be helpful to identify means to induce alternative cell death mode(s) that will overcome therapeutic resistance in these cancer cells.

Paraptosis, a type of programmed cell death (PCD) that is characterized by dilation of mitochondria and/or endoplasmic reticulum (ER), is independent of caspases and lacks apoptotic morphologies^4^. Although the molecular basis of paraptosis has not yet been extensively explored, this process is known to require *de novo* protein synthesis^5,6^. Various natural products, including curcumin^7^, celastrol^8^ and withaferin A^9^, have been shown to induce paraptosis in malignant breast cancer cells. In particular, curcumin induces paraptosis selectively in malignant breast cancer cells while sparing normal cells^7^. Moreover, curcumin-induced paraptosis is not blocked by the overexpression of various anti-apoptotic proteins. An understanding of cancer-selective action mechanism of curcumin could facilitate the development of safe and effective anti-cancer drugs, although its clinical application has been limited by its poor bioavailability. Recent studies have shown that paraptosis is associated with the generation of reactive oxygen species^8,10–13^, imbalances in the homeostasis of ions (e.g., Ca^2+^ and K^+^)^10,13–17^, and perturbation of cellular proteostasis via proteasomal inhibition and disruption of sulfhydryl homeostasis^8,10,13–15,18–20^. However, the mechanisms underlying paraptosis, particularly the signals responsible for the dilation of mitochondria and the ER, are still not clearly understood. Clarification of the genes critical for controlling paraptosis may suggest novel therapeutic targets for various diseases, including TNBCs, for which we currently lack effective druggable targets.

Over the past 20 years, major technological advances have yielded systems that can perform automated microscopic screening for visual phenotypes in cells and organisms. Various screening approaches have been used to evaluate anti-cancer and anti-viral efficacies for drug discovery, and these strategies have been adapted to 96-well or 384-well microtiter plate formats to enable low- to high-throughput applications^21^. One of the most powerful tools available for the rapid identification of new target genes that may act against malignant cancer cells is high-content screening (HCS), which combines high-throughput screening (HTS) with the ability to collect cellular images of biological processes. HCS has been used to characterize gene functions in cells subjected to RNAi or genetic perturbations, and to identify potential drug candidates from large libraries of small molecules^22^. Historically, HCS has often relied on relatively simple assays, such as assessment of cell growth/ viability or the levels of luminescent reporter genes. However, HCS may be performed using various visual profiling approaches, such as tagging a protein of interest with various-colored fluorescent protein or engineering a cell line to respond to stimuli appropriately^22,23^. The measurement of multiple parameters and the integration of these data at the single-cell level can enable researchers to perform complex tasks, such as precisely identifying the proteins involved in a specific biological process or predicting the target of a drug candidate^24,25^. For the successful HCS screening of the target candidates, however, it is critical to establish a specific and powerful assay concept.

The morphological features (dilation of mitochondria and the ER) of paraptosis have been characterized, but the biochemical features of this process are not well understood. A large-scale screening of genes that contribute to the phenotypic changes seen during paraptosis should add to our knowledge of the molecular basis underlying this novel cell death mode. In an attempt to identify the key regulators of paraptosis, we herein conceptualized a cell-based assay for detecting the mitochondrial changes that represent a main morphological feature of paraptosis. We used a multiparametric readout to simultaneously monitor: 1) the phenotypic changes of mitochondria during the curcumin-induced paraptosis of malignant breast cancer cells; and 2) the viability of these cells. This allowed us to rapidly score the effects of siRNAs from a siRNA library of 2,732 druggable genes. Our screening identified hits that were enriched for several classified signal pathways, suggesting that our method could be used to suggest new targets for anti-cancer therapies against malignant breast cancer cells. In addition, we show that USP10 may be a critical factor for curcumin-induced mitochondrial dilation and subsequent paraptotic cell death.

## Results

### Validation of the paraptosis model

**Figure 1:**
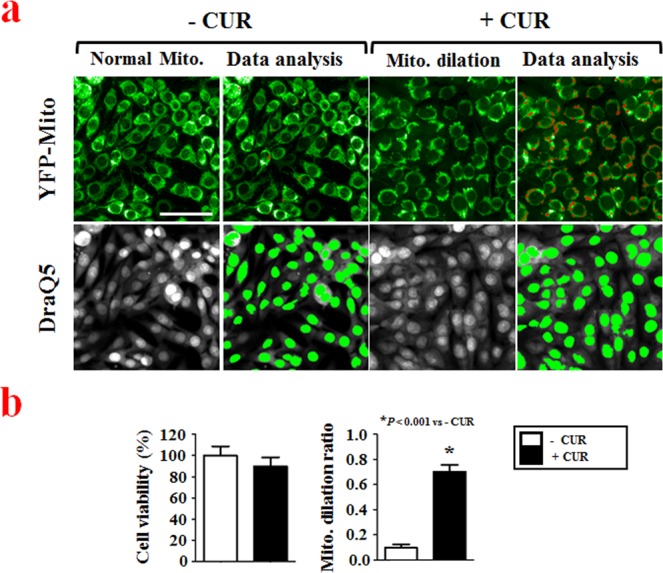
High-content image acquisition and data analysis. (**a**) YFP-Mito-expressing MDA-MB 435 S sublines (YFP-Mito cells) were cultured in 384-well plates at a density of 4,000 cells per well and treated with vehicle (0.5% DMSO (v/v); untreated control (− CUR)) or 40 μM curcumin for 12 h. The plates were fixed and stained with DraQ5, as described in the Materials and Methods. Images were acquired using an Opera automated laser confocal microscope system, with the green channel used for YFP-expressing mitochondria and the red channel used to detect DraQ5-stained nuclei. Bar, 50 μm. Image-based assay for phenotypic profiling of both cell viability and mitochondrial dilation was performed. The images were acquired from Columbus 2.3 software. (**b**) Cell viability and mitochondrial dilation ratios were quantitatively measured using the Columbus 2.3 software, as described in the Materials and Methods. ^*^P < 0.001 vs. untreated control (− CUR).

A main feature of paraptosis is an extensive cytoplasmic vacuolization that is derived from mitochondria and the ER^4^. We previously showed that curcumin induces paraptosis via formation of megamitochondria due to fusion of swollen mitochondria, and expansion of the ER structure by fusion of swollen ER components^7,8,15^ To validate our intended model, we confirmed the formation of megamitochondria in MDA-MB 435 S cells treated with curcumin by electron and confocal microscopy (Supplementary Fig. S1). The ER expansion seen during paraptosis is thought to arise due to an overload of misfolded proteins within the ER lumen and a subsequent increase of osmotic pressure, which draws water from the cytoplasm to distend the ER lumen^13,26^. However, the signals responsible for triggering mitochondrial dilation have not yet been clearly determined. To identify key regulators of paraptosis, we designed an image-based cellular screening assay that detects mitochondrial dilation, which is an initial and major morphological change in paraptosis, in cells exposed to a small interfering RNA (siRNA) library. For this purpose, we employed MDA-MB 435 S sublines stably expressing a YFP-Mito construct (YFP-Mito cells). We speculated that the fluorescence microscopy would enable us to clearly distinguish the megamitochondria (mitochondrial dilation) formed during the paraptosis of YFP-Mito cells from the elongated mitochondria of untreated cells. If this was the case, detecting the extent of mitochondrial dilation could allow us to perform direct imaging and quantification using an automated fluorescence microscope. Using a high-content approach, we scored the effects of transfected siRNAs using a multiparametric readout: we detected both the progression of paraptosis (by the amount of detected mitochondrial dilation) and cytotoxicity (by the relative number of nuclei in treated versus untreated cells). To verify the concept of our assay, we used an Opera automated laser confocal microscope to observe the distribution of YFP-expressing mitochondria in cells treated with vehicle (0.5% DMSO, v/v) or 40 μM curcumin for 12 h. Using the analysis module of the Columbus 2.3 HCS analysis software, we first identified the nucleus of each cell using an object-recognition tool based on the pixel gradient of the red DraQ5 channel (Fig. 1a). To assess mitochondrial dilation, we designed an assay to detect the total spot number based on the area and intensity of the fluorescence following treatment of cells with 40 μM curcumin in green channel. The negative control showed the elongated and branched YFP signal pattern of normal mitochondria, whereas curcumin treatment drastically induced mitochondrial dilation (Fig. 1a,b). These results indicate that our image-based cellular assay system could be used to evaluate the activities of candidate factors that might regulate paraptosis.

### Assay adaptation and validation

As a first step toward automated HTS, we miniaturized our assay from a 24-well format to a 384-well microtiter plate format, and assessed whether curcumin induced mitochondrial dilation under the siRNA-supplemented screening condition. By reverse transfection, non-targeting siRNA was dispensed into 384-well microtiter plates and then YFP-Mito cells were seeded and incubated for 48 h. The seeding density was 4,000 cells per well, as preliminary experiments indicated that this density yielded a good overall consistency of cell distribution (data not shown). We then treated cells with serially diluted curcumin (8 doses between 2.5 μM and 60 μM) for 24 h, and assessed cytotoxicity and mitochondrial dilation. Under the tested conditions, curcumin dose-dependently induced both mitochondrial dilation and cell death (Supplementary Fig. S2). Our results demonstrated that our mitochondrial dilation assay to measure paraptosis could be adapted to a 384-well microtiter plate format, giving us the confidence to move forward in developing this concept as an automated high-content screening assay.

Recent studies have shown that mitochondria and the ER are interconnected physically and functionally by the mitochondria-associated ER membrane (MAM) structure, which forms during the progression of paraptosis^14^. In addition, we recently showed that paraptotic inducers (e.g., curcumin, dimethoxycurcumin and celastrol) inhibit proteasome activity and upregulate CHOP, which is a member of the C/EBP family of transcription factors^7,8,15^. In the present study, we first evaluated whether CHOP siRNA could be used as a positive control for blocking curcumin-induced paraptosis in our assay using the 384-well format.

**Figure 2:**
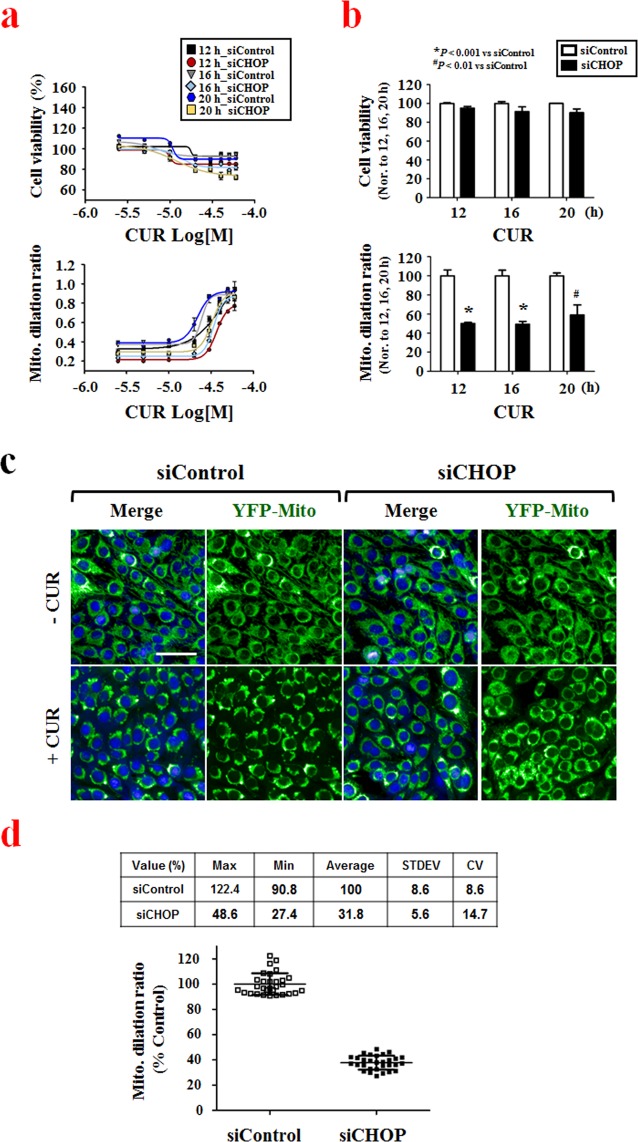
Adapting the assay to the 384-well microtiter plate format. (**a**) YFP-Mito cells were transfected with non-targeting siRNAs or CHOP siRNAs and further treated with the indicated concentrations of curcumin for 12, 16 and 20 h. Cell viability or the mitochondrial dilation ratio were quantitatively measured using the Columbus 2.3 software. (**b**) Quantitation of cell viability and mitochondrial dilation ratio. Cell viability or mitochondrial dilation ratios obtained after the indicated durations of 30 μM curcumin treatment were normalized with respect to controls (non-targeting siRNA (siControl)- transfected cells treated with 30 μM curcumin for the respective time points), as measured using the Columbus 2.3 software. ^*^P < 0.001 vs. siControl; ^#^P < 0.01 vs. siControl. (**c**) YFP-Mito cells were transfected with non-targeting siRNAs or CHOP siRNA and further treated with 30 μM curcumin for 12 h. Cells were observed under an Opera microscope system. Bar, 50 μm. (**d**) YFP-Mito cells were transfected with either 20 nM non-targeting siRNAs (negative control) or 20 nM CHOP siRNA (positive control), incubated for 48 h, and treated with 30 μM curcumin for 12 h. Mitochondrial dilation ratios of control wells were assessed by column scatter plot and quantitatively measured using the Columbus 2.3 software.

To determine the optimal assay conditions for the screening to detect mitochondrial dilation, we treated YFP-Mito cells with serially diluted curcumin for 12, 16 or 20 h, and measured cell viability and the mitochondrial dilation ratio (Fig. 2a,b). Since mitochondrial dilation precedes overt paraptotic cell death, we searched for an assay condition under which we could observe obvious mitochondrial dilation without induction of cell death. We found that treatment of cells with 30 μM curcumin for 12 h induced mitochondrial dilation without cytotoxicity, and that this was effectively inhibited by transfection of CHOP siRNA (Fig. 2b,c), which efficiently knocked down CHOP expression (Supplementary Fig. S3). Thus, we used this treatment as the optimal assay condition to subsequently screen our siRNA library to identify key regulators of paraptosis.

Next, we evaluated the assay performance in a control run using three 384-well microtiter plates containing cells transfected with the negative (non-targeting siRNA) or positive (CHOP siRNA) control siRNAs and further treated with 30 μM curcumin or vehicle (0.5% DMSO v/v). The assay had normalized mitochondrial dilation ratio; the average high mitochondrial dilation ratio was (100 ± 8.6)% with 8.6% coefficient of variation (CV) and the average low mitochondrial dilation ratio was (31.8 ± 5.6)% with 14.7% CV (Fig. 2d). Given that a heterogeneous cellular response is typically seen in cell-based assays for siRNA screening^27^, these results showed that the performance of our assay model was excellent. In addition, as we expected, no change in cell viability (assessed by nuclei numbers) was detected in the negative or positive controls under the tested conditions (data not shown). Based on these data, we moved forward in developing our system for automated high-content screening.

### siRNA-based screen to identify genes involved in curcumin-induced paraptosis

**Figure 3:**
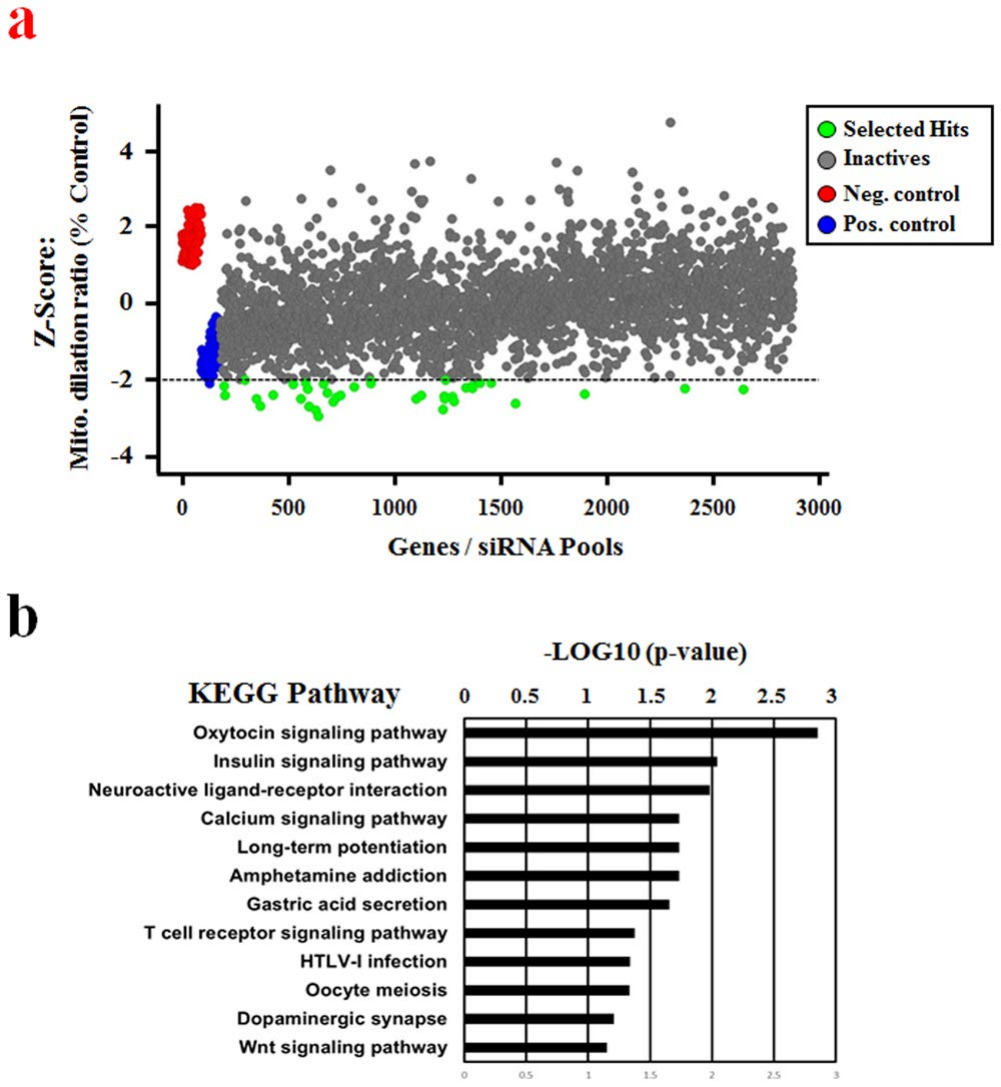
Results of screening for curcumin-induced paraptotic regulators. Assay sensitivity and performance were evaluated against a library containing 2,732 siRNAs. (**a**) Scatter plot analysis of the primary HCS screening results for 2,732 siRNAs: the x-axis indicates the order of the respective gene, and the y-axis shows the Z-scores of the percent relative mitochondrial dilation ratio. Thirty-eight siRNAs were selected as having a mitochondrial dilation ratio < −2 Z-score and a relative cell number above 80% when normalized to non-targeting siRNA (siControl)-transfected samples. Genes that induced cytotoxicity were eliminated from the scatter list. (**b**) Enrichment of the selected hits from the primary screen. Hit lists generated from the hit selection process were analyzed for pathway enrichment using the KEGG pathway database. Pathways with expectation values < 0.05 and a gene-set overlap count of three or greater are shown.

To identify genes involved in curcumin-induced paraptosis, we optimized our newly adapted assay for HTS use, as depicted in Table 1, and tested it against a human druggable subset library containing 2,732 individual siRNAs, including siRNAs against various ion channels, proteases, phosphatases, kinases, ubiquitin conjugation factors and GPCRs. This siRNA library was plated in nine 384-well microtiter plates. Half of the wells of columns 1, 2, 23 and 24 contained 20 nM non-targeting siRNA (negative control), while the other half were loaded with 20 nM CHOP siRNA (positive control). To monitor the performance of our assay throughout the screen, we loaded columns 1 and 24 with 0.5% DMSO (v/v, untreated control) and loaded columns 2 to 23 with 30 μM curcumin (treatment control to induce mitochondrial dilation). To examine the efficiency of each siRNA, the data for mitochondrial distributions and cytotoxicity were plotted in a scatter graph (Fig. 3a). For mitochondrial dilation, a positive response was defined using a Z-Score < −2 as a cutoff. Cytotoxic siRNAs were defined as those that reduced the number of nuclei by death-associated detachment from the plates and were filtered by setting an 80% threshold. This analysis eliminated seven of the cytotoxic siRNAs, including those against PLK1 and PPP1CC, from the set of candidate siRNAs and our multiparametric readout therefore yielded 38 candidate siRNAs (Supplementary Table S1). To obtain in-depth biological information on the selected hits, Kyoto Encyclopedia of Genes and Genomes (KEGG) database analyses were conducted using a DAVID online analysis tool. As shown in Fig. 3b, this analysis identified 12 significantly relevant pathways, including the oxytocin signaling pathway, calcium signaling pathway, long-term potentiation, amphetamine addiction, gastric acid secretion, HTLV-1 infection, oocyte meiosis and the Wnt signaling pathway, are associated with GPCR-related signaling.

**Table 1:** Workflow of curcumin-induced mitochondrial dilation assay.

Step	Parameter	Value	Description
1	Library siRNAs plating	1 μL	1 μM in siRNA Buffer
2	siRNA buffer mixture	4 μL	siRNA Buf.:Opti MEM = 1.5:2.5
3	Transfection reagent mixture	5 μL	Dharmafect1:Opti MEM = 1:50
4	Incubation time	20 min	Bio Safety Cabinet
5	Cell seeding	40 μL	4,000 YFP-Mito MDA-MB 435S cells in 10% FBS DMEM media w/o antibiotics
6	Incubation time	48 h	37 °C, 5% CO_2_
7	Mitochondrial dilation	0.25 μL	30 μM Curcumin
8	Incubation time	12 h	37 °C, 5% CO_2_
9	Nuclei staining	10 μL	10 μM DraQ5 in 1 X PBS for 10 min
10	Assay readout	605 nm/690 nm and 488 nm/535 nm (ex/em)	Opera microscope system
11	Image analysis	—	Multiparametric analysis using Columbus software

### USP10 effectively inhibits curcumin-induced paraptosis in malignant breast cancer cells

**Figure 4:**
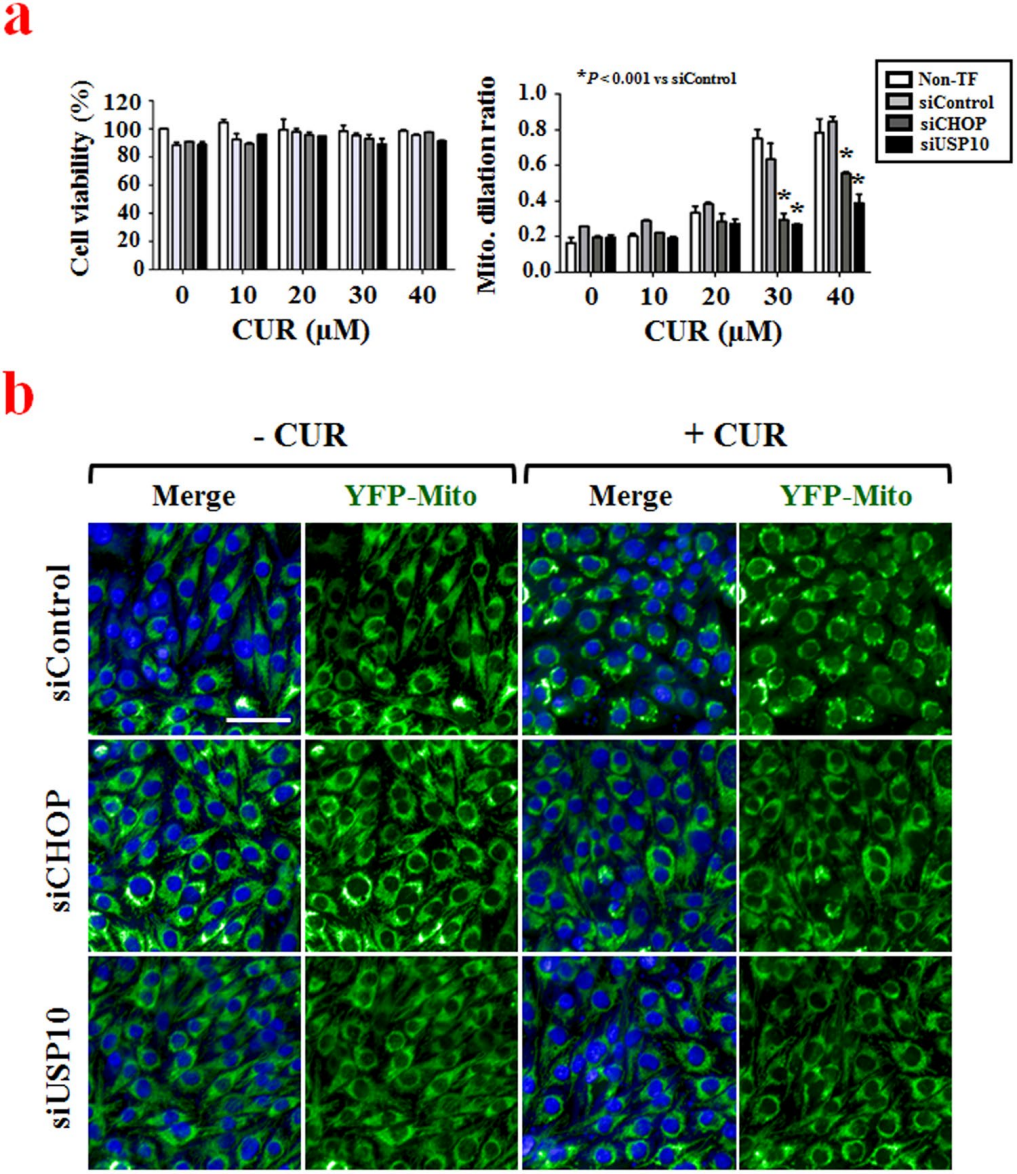
Secondary screening to confirm the involvement of USP10 in curcumin-induced mitochondrial dilation. (**a**) YFP-Mito cells were transfected with non-targeting siRNAs, CHOP siRNAs or USP10 siRNAs and further treated with the indicated concentrations of curcumin for 12 h. Viability and mitochondrial dilation ratios were quantitatively measured using the Columbus 2.3 software. ^*^P < 0.001 vs. siControl. (**b**) YFP-Mito cells were transfected with non-targeting siRNA, CHOP siRNAs or USP10 siRNA and further treated with 30 μM curcumin for 12 h. Cells were observed under an Opera microscope system. Bar, 50 μm.

**Figure 5:**
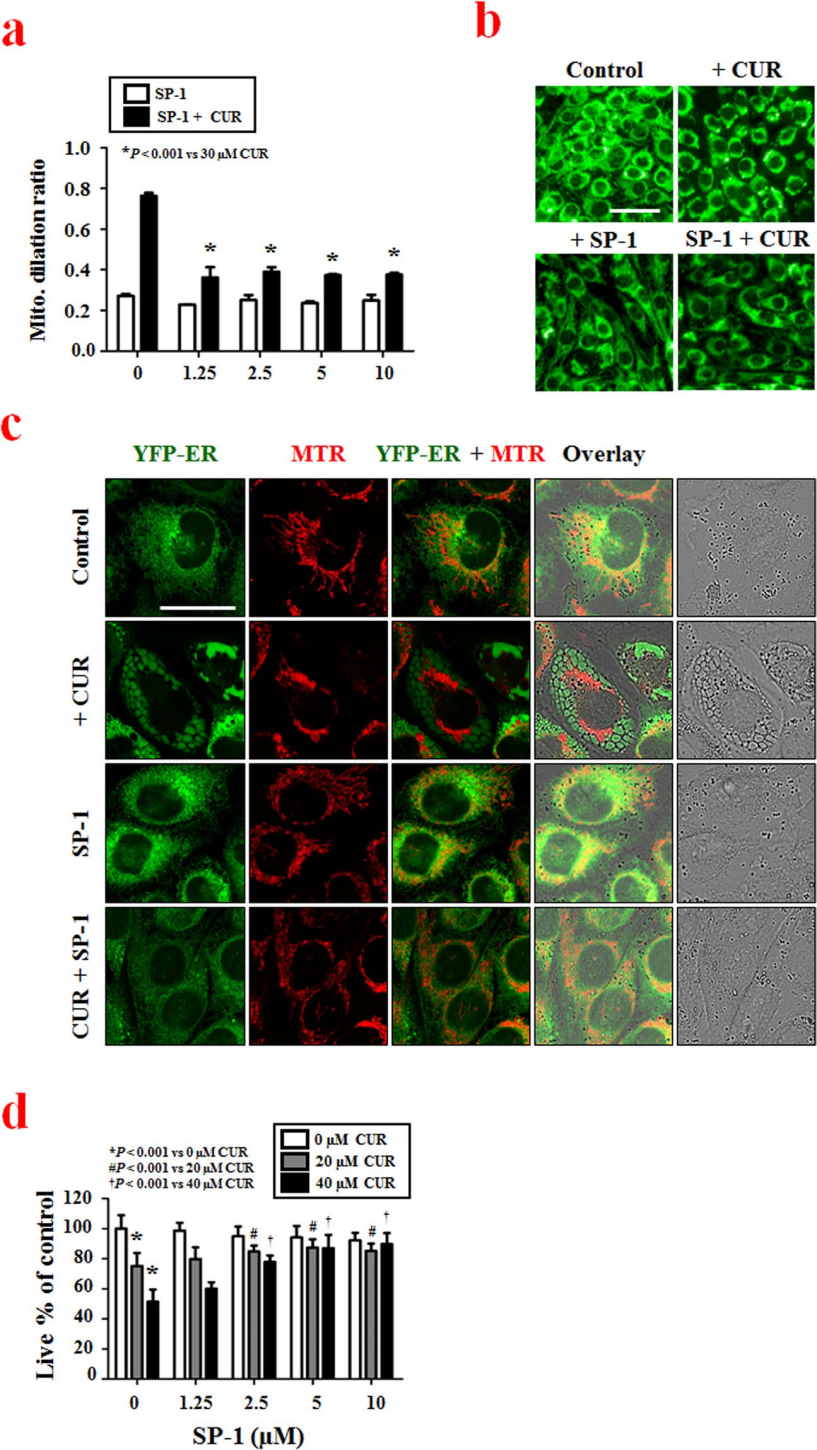
The USP10 inhibitor, spautin-1, effectively inhibits curcumin-induced paraptosis in malignant breast cancer cell. (**a**) YFP-Mito cells were pretreated with the indicated concentrations of spautin-1 and further treated with 30 μM curcumin for 12 h. Mitochondrial dilation ratios were quantitatively measured using the Columbus 2.3 software. ^*^P < 0.001 vs. 30 μM curcumin. (**b**) YFP-Mito cells were treated with 5 μM spautin-1 and/or 30 μM curcumin for 12 h. Cells were observed under an Opera microscope system. Bar, 50 μm. (**c**) YFP-ER cells were treated with 5 μM spautin-1 and/or 40 μM curcumin, stained with 200 nM MitoTracker Red (MTR) for 20 min and observed by confocal microscopy. Bar, 30 μm. (**d**) MDA-MB 435 S cells were pretreated with the indicated concentrations of spautin-1 and further treated with 20 or 40 μM curcumin for 24 h. Cellular viability was assessed using calcein-AM and EthD-1. ^*^P < 0.001 vs. 0 μM curcumin; ^#^P < 0.001 vs. 20 μM curcumin; ^†^P < 0.001 vs. 40 μM curcumin.

**Figure 6:**
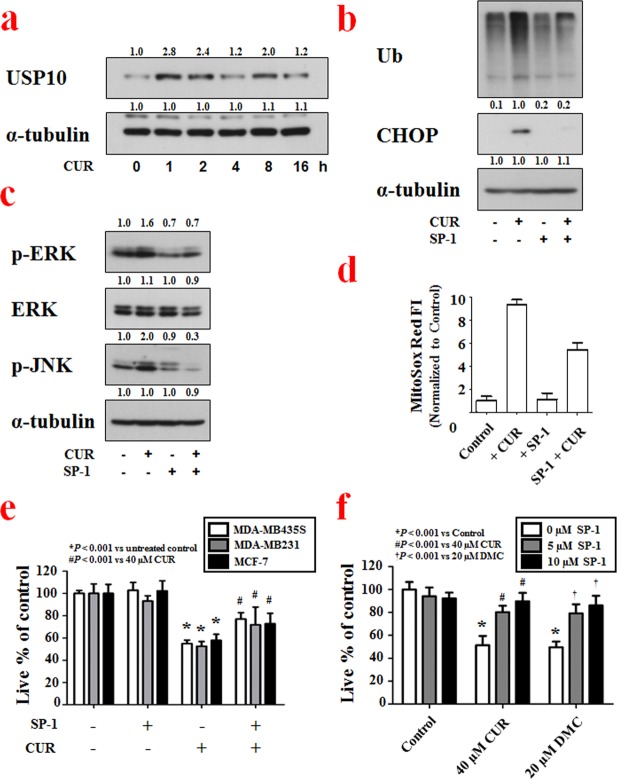
Spautin-1 effectively inhibits curcumin-induced-paraptotic signaling in malignant breast cancer cell. (**a**) MDA-MB 435 S cells treated with 40 μM curcumin for the indicated time points were subjected to Western blotting of USP10, with α-tubulin detected as a loading control. Full-length blots are presented in Supplementary Fig. S7. (**b,c**) MDA-MB 435 S cells treated with 10 μM spautin-1 and/or 40 μM curcumin (**b**) for 8 h or (**c**) for 4 h were subjected to Western blotting of the indicated proteins, with α-tubulin detected as a loading control. Full-length blots are presented in Supplementary Fig. S7. (**d**) 40 μM curcumin and or 5 μM spautin-1 treated cells were stained with MitoSox Red and subjected to flow cytometry. (**e**) Cells were treated with 5 μM spautin-1 and/or 40 μM curcumin for 24 h. Cellular viability was assessed using calcein-AM and EthD-1. ^*^P < 0.001 vs. untreated control; ^#^P < 0.001 vs. 40 μM curcumin. (**f**) MDA-MB 435 S cells were pretreated with 5 or 10 μM spautin-1 and further treated with 40 μM curcumin or 20 μM DMC for 24 h. Cellular viability was assessed using calcein-AM and EthD-1. ^*^P < 0.001 vs. Control; ^#^P < 0.001 vs. 40 μM curcumin; ^†^P < 0.001 vs. 20 μM DMC.

Using the optimized assay conditions and established workflow (Table 1), we successfully screened for siRNAs that altered curcumin-induced mitochondrial dilation in YFP-Mito cells and identified genes that may be important to this process. To confirm whether our selected hits demonstrated target-dependent specificity, we set out to validate one of the identified candidate gene products. We focused our attention on USP10 (ubiquitin specific peptidase 10, which specifically cleaves ubiquitin from ubiquitin-conjugated protein substrates) by the following reasons. First, the USP10 siRNA demonstrated the strongest and most consistent effects in rescuing curcumin-induced mitochondrial dilation in our cell-based siRNA screening. Second, a specific inhibitor of USP10, sparutin-1, is available to test the involvement of USP10 in mitochondrial dilation during paraptosis. To confirm that USP10 knockdown inhibits curcumin-induced mitochondrial dilation, we compared the effect of USP10 siRNA with that of CHOP siRNA, which was used as a positive control in our screening, on curcumin-induced mitochondrial dilation. When we transfected YFP-Mito cells with USP10 siRNA and then applied various concentrations (0–40 μM) of curcumin, we found that USP10 knockdown inhibited mitochondrial dilation more effectively than CHOP knockdown (Fig. 4a). Representative mitochondrial dilation results obtained from CHOP- or USP10-knockdown cells treated with 30 μM curcumin are shown in Fig. 4b and Supplementary Figure S4. The importance of USP10 in curcumin-induced paraptosis was further confirmed by using its inhibitor, spautin-1. While treatment of YFP-Mito cells with 30 μM curcumin for 12 h induced mitochondrial dilation, pretreatment with spautin-1 significantly and dose-dependently inhibited the curcumin-induced morphological changes of mitochondria (Fig. 5a,b). Since another morphological feature of paraptosis is ER dilation, we next tested whether spautin-1 pretreatment also affected curcumin-induced ER dilation. We did this by staining YFP-ER cells with MitoTracker-Red (MTR). While treatment of YFP-ER cells with curcumin for 12 h induced dilation of both mitochondria and the ER, spautin-1 pretreatment effectively inhibited these dilations (Fig. 5c). In addition, spautin-1 pretreatment dose-dependently inhibited the cell death seen in MDA-MB 435 S cells treated with curcumin (Fig. 5d). These results indicate that USP10, which was selected from our image-based HCS as a potential effector of curcumin-induced paraptosis, may play a key role in this process. When we examined the expression of USP10 following curcumin treatment, interestingly, USP10 protein levels oscillated, with a first peak seen at 1 h of curcumin treatment and a second peak detected at 8 h (Fig. 6a). We next investigated whether the upregulation (and thus increased activity) of USP10 plays a critical role in curcumin-induced paraptosis by affecting the key signals involved in this process. We previously showed that curcumin treatment triggers the accumulation of poly-ubiquitinated proteins and CHOP, and that this upregulation of CHOP is critically involved in curcumin-induced paraptosis, particularly in the context of ER dilation^7^. We also showed that ERK activation, JNK activation, and the generation of mitochondrial superoxide critically contribute to curcumin-induced paraptosis^7^. When we tested the effects of spautin-1 on these important signals associated with curcumin-induced paraptosis, we found that spautin-1 pretreatment effectively blocked the curcumin-induced upregulation of poly-ubiquitinated proteins and CHOP (Fig. 6b), blocked the curcumin-induced activation of ERKs and JNKs (Fig. 6c), and reduced the curcumin-induced generation of mitochondrial superoxide (Fig. 6d). Furthermore, pretreatment with 5 μM spautin-1 significantly attenuated the cell death induced by curcumin not only in MDA-MB 435 S but also in other breast cancer cells, including MDA-MB 231 and MCF-7 cells (Fig. 6e, Supplementary Fig. S5a). We recently showed that dimethoxycurcumin (DMC), a curcumin derivative in which the phenolic-OH groups of curcumin are replaced with methoxy groups^10,11^, induces paraptosis more potently than curcumin in malignant breast cancer cells^15^. Therefore, we further examined whether spautin-1 could also inhibit DMC-induced paraptosis. Indeed, we found that pretreatment with spautin-1 dose-dependently inhibited the death as well as vacuolation induced by 20 μM DMC in MDA-MB 435 S cells (Fig. 6f, Supplementary Fig. S5b). Taken together, our results indicate that USP10, a hit selected from our HCS for potential effectors of mitochondrial dilation, may act as an early key signal in curcumin-induced paraptosis.

## Discussion

Malignant cancer cells are often genetically heterogeneous and resist the induction of apoptosis^28,29^. Accumulating evidence suggests that the effective elimination of malignant cancer cells may require not only classical apoptotic pathways (mitochondrial and/or death-receptor pathways) but also non-apoptotic pathways^4^. In addition, researchers have speculated that the relative sensitivity of cancer cells (versus normal cells) to oxidative and ER stress could be exploited for the rational design of cancer therapeutics^17,30,31^. Thus, induction of paraptosis, which is a cell death mode that targets both mitochondria and the ER, may offer an attractive therapeutic strategy to effectively kill malignant cancer cells that resist conventional pro-apoptotic cancer therapies. We previously reported that curcumin induces extensive swelling and fusion of mitochondria leading to formation of megamitochondria (Supplementary Fig. S1)^7^, and subsequent expansion of ER-derived vacuoles^7^, resulting in an irreversible cell death. However, the signals responsible for the dilation of mitochondria and the ER are still not clearly understood. Here, in an effort to decipher the key signals responsible for mitochondrial dilation, an initial key event of curcumin-induced paraptosis, we designed and performed a multiparametric siRNA screen intended to identify genes involved in paraptosis.

As the screening strategy, we exploited multiple readouts to find the most biologically relevant targets in curcumin-induced paraptosis: we examined mitochondrial dilation by using breast cancer cells that expressed fluorescence selectively in mitochondria (YFP-Mito cells), and we measured cell viability. Cellular morphologies were examined after transfection with siRNAs and further treatment with curcumin for 12 h. The resulting dual-readout assay was easily implemented for the screening of a druggable subset siRNA library that comprised sub-libraries of ion channels, proteases, phosphatases, kinases, ubiquitin conjugation proteins, and GPCRs. In our screening system, the siRNAs of interest could be rapidly identified by their ability to regulate curcumin-induced paraptosis (i.e., decrease curcumin-induced mitochondrial dilation) without generating cytotoxicity. To validate our screening method and hit-selection strategy, we performed KEGG pathway analysis of selected hits. We found that many of the identified genes were associated with GPCR signaling, including those encoding components of oxytocin signaling, neuroactive ligand-receptor interactions, calcium signaling, long-term potentiation and dopaminergic synapses (Fig. 3b), suggesting that GPCR-associated signals may be critically linked to curcumin-induced paraptosis. We also note that oxytocin receptor expression has been observed in cases of primary and metastatic carcinomas of breast^32^ and that oxytocin was shown to inhibit the proliferation of human breast cancer cell lines^33^ and decrease the tumor growth of rat and mouse mammary carcinomas^34^. This suggests that our screening successfully identified the genes that are enriched in the pathways of interest, not just a random set of genes.

In the present study, the siRNA corresponding to USP10, a member of the ubiquitin-specific protease family of cysteine proteases, showed one of the strongest abilities to rescue curcumin-induced mitochondrial dilation. Ubiquitin-specific proteases (USPs) are one class of deubiquitinases (DUB) that targets several key proteins involved in regulation of tumorigenesis, apoptosis, senescence, and autophagy^35^. They hydrolyze the amide bond between the C-terminal glycine residue of ubiquitin and primary amino groups of the substrate protein. Distinct USPs including more than 50 members may have specialized functions^36,37^, although their detailed action mechanisms are not yet fully understood. Consistent with our screening results on the effect of USP10 siRNA, pretreatment with spautin-1, a specific inhibitor of USP10, markedly inhibited curcumin-induced morphological changes of mitochondria and subsequent paraptotic cell death in various breast cancer cells. In addition, spautin-1 pretreatment effectively inhibited the paraptosis induced by not only curcumin but also dimethoxycurcumin, a derivative of curcumin with a more potent anti-cancer effect^15^. Furthermore, we found that spautin-1 pretreatment effectively inhibited curcumin-induced dilation of the ER, which is another morphological marker of paraptosis^7^. Moreover, spautin-1 pretreatment potently inhibited all of the tested key signals involved in curcumin-induced paraptosis, including the accumulation of poly-ubiquitinated proteins and CHOP, the activation of ERKs and JNKs, and the generation of mitochondrial superoxide. Taken together, these results suggest that USP10 may act as an early key signal in curcumin-induced paraptosis.

Spautin-1 was initially identified as an inhibitor of both USP10 and USP13^38^. Although an siRNA against USP13 was included in the human druggable library set that we screened for key factors of curcumin-induced paraptosis, our analysis did not select USP13 as an accelerating or inhibiting hit for curcumin-induced paraptosis. In addition, both spautin-1 and the USP10 siRNA effectively inhibited curcumin-induced mitochondrial dilation. These observations suggest that USP13 may not be involved in curcumin-induced paraptosis, and the inhibiting effect of spautin-1 on curcumin-induced paraptosis may be due to inhibition of USP10. Recent studies found that USP10 deubiquitinates and stabilizes the tumor suppressor, p53^18^, thereby contributing to the DNA damage response^18^. In addition, AMPK^39^ and Beclin-1^38^ have been proposed as the substrates of USP10-mediated deubiquitination. Interestingly, upon exposure to an oxidant, USP10 regulates cell death via its antioxidant activity without any requirement for its deubiquitinase activity^20^, suggesting that USP10 may have both deubiquitinase-dependent and -independent anti-stress functions depending on the stimulus and cellular context. When we treated p53-mutant MDA-MB 435 S cells^40^ with spautin-1 and/or curcumin for 4 h, we found no marked alteration in the protein levels of p53 or beclin-1 (Supplementary Fig. S6). AMPK phosphorylation was slightly increased by curcumin, but spautin-1 pretreatment did not dramatically affect this curcumin-induced AMPK phosphorylation. When we examined the long-term effect of spuatin-1 and/or curcumin by extending the treatments to 24 h, we found that the short- and long-term treatments both failed to alter the protein level of beclin-1. However, the protein levels of USP10 and p53 were markedly reduced by spautin-1 treatment for 24 h; this is consistent with the results previously reported by Yuan J. *et al*.^18^ Interestingly, the protein level of p53 was also reduced by treatment with curcumin alone or spautin-1/curcumin. Extended exposure of our Western blot for p53 revealed that high-molecular-weight p53 accumulated under curcumin treatment and accumulated further under spautin-1 cotreatment. These results suggest that the proteasome-inhibiting activity of curcumin might lead to the accumulation of poly-ubiquitinated p53, and that this is further enhanced by spautin-1 co-treatment. The phosphorylation level of AMPK was increased by either curcumin or spautin-1, and this level showed a slight additional increase in cells subjected to curcumin/spautin-1 treatment. Our results indicate that curcumin-induced p53 downregulation and AMPK phosphorylation are not counteracted by spautin-1 treatment. This suggests that the critical role of USP10 in curcumin-induced paraptosis does not act via the modulation of beclin-1, p53 or AMPK, and that there are yet-unidentified targets of USP10 in curcumin-induced paraptosis. Additional work will be needed to investigate the detailed underlying mechanism by which USP10 contributes to paraptotic cell death in malignant breast cancer cells. Additional work is needed to investigate the detailed underlying mechanism by which USP10 contributes to mitochondrial dilation and subsequent paraptotic cell death in malignant breast cancer cells.

In summary, we herein configured a robust and efficient high-content screening method for curcumin-induced paraptosis by combining automated microscopy with HTS technology. Our screening method simultaneously monitors the cytotoxicity of the tested genes and assesses the fluorescence level to detect changes in the mitochondrial phenotype. We miniaturized this assay to a 384-well microtiter plate format to facilitate the screening of chemical libraries, and identified several signaling pathways that may be involved in dilation of mitochondria in malignant breast cancer. We believe that this assay concept could be further developed to detect phenotypic changes in the ER, which would enable the identification of genes that are critically involved in ER dilation. This would further contribute to our understanding of the signaling networks of paraptosis, which is a novel cell death mode whose biochemical features have not yet been clearly delineated. In addition, high-content screening using our systematic assay concept may contribute to the identification of potential targets and the development of new therapeutic strategies for selectively killing malignant breast cancer cells.

## Materials and Methods

### Chemicals, antibodies and cell culture

Curcumin, dimethoxycucumin (DMC), Spautin-1 and Dimethyl sulfoxide (DMSO) were purchased from Sigma (St. Louis, MO). DraQ5 (BioStatus, Leicestershire, UK). Dharmafect 1 transfection reagent, 5× siRNA buffer and RNase free water were purchased from GE Dharmacon (Lafayette, CO). MitoTracker Red and MitoSox Red were from Molecular Probes (Eugene, OR, USA). We used antibodies against α-tubulin and ubiquitin (Santa Cruz biotechnology, Santa Cruz, CA); phospho-ERK1/2, total ERK1/2, phospho-JNK, CHOP (Cell Signaling, Beverly, MA); USP10 (Abcam, Cambridge, MA); HRP-conjugated anti-rabbit IgG and anti-mouse IgG (Invitrogen, San Diego, CA). MDA-MB 435 S, MDA-MB 231 and MCF-7 human breast cancer cell lines, which were purchased from American Type Culture Collection (ATCC, Manassas, VA), were cultured in 5% CO_2_ at 37 °C using DMEM supplemented with 10% fetal bovine serum (FBS) and 1% antibiotics GIBCO-BRL, Grand Island, NY). Cell culture passage number less than ten was used in the present study.

### Establishment of the stable cell lines expressing the fluorescence specifically in mitochondria or the ER

To establish the stable cell lines expressing the fluorescence specifically in mitochondria or the ER, MDA-MB 435 S cells were transfected with the pEYFP-Mito vector or pEYFP-ER, respectively (Clontech Laboratories, Mountain View, CA). Stable cell line overexpressing pEYFP-Mito (YFP-Mito cells) and pEYFP-ER (YFP-ER cells) were selected with fresh medium containing 500 μg/mL G418 (Invitrogen, San Diego, CA). the morphological changes of the ER and mitochondria were observed under a K1-Fluo confocal laser-scanning microscope (Nanoscope Systems. Daejeon, Korea).

### Liquid dispensation and automation

For the plating and transfer of siRNAs, we used a custom-designed 384-head pipette system with disposable low-volume polypropylene tips on a CyBi®-Well platform (CyBio, Woburn, MA). The addition of cells, dispensation of growth media, mixing steps during compound treatment or siRNA transfection, fixation and staining of cells were performed using a Multidrop 384 pipette (Thermo Fisher Scientific, Waltham, MA) for assay adaptation or a WellMate (Thermo Fisher Scientific) for screening. The assay plates were incubated in a Steri-Cult Incubator (Sanyo, Japan) under controlled humidity and 5% CO_2_ at 37 °C.

### Screening of the siRNA library that modulates curcumin-induced paraptosis

We used an OTP (On-TARGET plus) human druggable subset library containing 2,732 individual siRNA duplexes (GE Dharmacon, Lafayette, CO). This collection consists of sub-libraries representing ion channels, proteases, phosphatases, kinases, ubiquitin conjugation-related factors, and G-protein coupled receptors (GPCRs). siRNA library stocks were formulated in siRNA buffer (GE Dharmacon) at 1 μM, and were added to 4% of the final assay volume for a final siRNA concentration of 20 nM. For siRNA reverse transfection, 1 μl of 1 μM siRNAs per well were dispensed to a 384-well microtiter plate (Greiner Bio-one, Frickenhausen, Germany) using the Cybi-Well, and 1× siRNA buffer was dispensed at 4 μl per well using the WellMate. Immediately thereafter, a 0.1:4.9 mixture of transfection (TF) reagent mixture and Opti-MEM (GIBCO-BRL, Grand Island, NY) was dispensed to the plate at 5 μl per well using the WellMate. The plate was incubated for 20 min, and then YFP-Mito cells were seeded at 4,000 cells per well in 40 μl of FBS-containing media without antibiotics, using the WellMate. The plate was incubated for 48 h and then loaded with 0.25 μl/well curcumin (final concentration, 30 μM in 0.5% DMSO, v/v). After incubation for 12 h, the cells were fixed for 30 min with 4% paraformaldehyde (PFA, w/v) and nuclei were stained for 1 h with DraQ5 (BioStatus). Images of YFP-Mito signals and the nuclei were acquired using an Opera microscope system (Perkin Elmer, Waltham, MA) and analyzed with the Columbus 2.3 software (Perkin Elmer).

### Image acquisition, data analysis, and management of screening data

For assay development and screening, images were acquired using the Opera automated laser confocal microscope system. Images were captured at the following wavelengths: 605 nm excitation/690 nm emission in the red channel for DraQ5-stained nuclei, and 488 nm excitation/540 nm emission in the green channel for YFP-expressing mitochondria. The exposure times were 80 and 40 ms, respectively. Nine images per well were collected using a 20× magnifying objective. Images were analyzed using the segmentation algorithm built into the Columbus 2.3 software; this algorithm counts nuclei as the number of objects with DraQ5 pixel intensities above background and counts dilated mitochondria spots as the number of objects with YFP pixel intensities above background. Since the nuclei of dead cells are removed during the fixation and staining steps of the screening process, cell viability was measured as the percentage of nuclei counted in treated versus untreated control cells. The mitochondrial dilation ratio was calculated as the number of total mitochondrial dilation spots/total cell number.

### Pathway analysis based on the selected siRNAs

To explore the pathway enrichment of the screened genes, we conducted KEGG (Kyoto Encyclopedia of Genes and Genomes) database analyses using the DAVID (the Database for Annotation, Visualization and Integrated Discovery) online analysis tool.

### Measurement of cellular viability using calcein-AM and EthD-1

Cells (5 × 10^4^ cells) were cultured in 24-well plates and treated as indicated. For measurement of cellular viability, 2 μM calcein-AM, a green fluorescent indicator of the intracellular esterase activity of cells, and 4 μM EthD-1, a red fluorescent indicator of membrane damaged (dead) cells, were added to each well, and the plates were incubated for 5 min in 5% CO_2_ at 37 °C. Cells were then observed under a fluorescence microscope (Axiovert 200 M, Carl Zeiss) equipped with Q2 Zeiss filter sets #46 and #64HE. Viable cells, corresponding to those that exclusively exhibited green fluorescence, were counted in five fields per well at ×200 magnification. Only exclusively green cells were counted as live because bicolored (green and red) cells cannot be unambiguously assigned to live or dead groups. The percentage of live cells, calculated as green cells/(green + red + bicolored cells), was normalized to that of untreated control cells (100%).

### Western blotting

Western blotting was performed as described previously^7^.

### Measurement of mitochondrial superoxide levels

To measure mitochondrial superoxide levels, treated cells were incubated with 2.5 μM MitoSOX Red at 37 °C for 20 min, washed with PBS, and analyzed immediately by flow cytometry using a FACSAria™ III.

### Statistical analysis

All hit confirmation-related data are presented as the mean ± S.D. (standard deviation) from at least three separate experiments. Statistical analysis was performed with the GraphPad Prism software (GraphPad Software Inc., Sandiego, CA) was used to perform the statistical analyses. Normality of data was assessed by Kolmogorov-Smirnov test and equal variance was assessed using Bartlett’s test. For normal distribution, statistical differences were determined using an analysis of variance (ANOVA) followed by Bonferroni’s multiple comparison test. P < 0.05 was considered statistically significant.

## Supplementary information

Supplementary information
