# Supplementary material for: RNAi Screening-based Identification of USP10 as a Novel Regulator of Paraptosis
Source: Sci Rep. 2019 Mar 20;9:4909. doi: 10.1038/s41598-019-40982-z (PMC6427038; doi:10.1038/s41598-019-40982-z)
Supplement: Supplementary file 1 — Supplementary information [file 41598_2019_40982_MOESM1_ESM.pdf]

Supplemental Information

RNAi Screening-based Identification of USP10 as a Novel Regulator of Paraptosis

Jin Yeop Kim[1,2], Dong Min Lee[1], Hyun Goo Woo[3], Ki Deok Kim[2], Hong Jae Lee[1], Yong-Jun Kwon[2,4,*] and Kyeong Sook Choi[1,*]

[1]Department of Biochemistry, Department of Biomedical Sciences, Ajou University School of Medicine, Suwon, Korea

[2]Institut Pasteur Korea, 696 Sampyeong-dong, Bundang-gu, Gyeonggi-do, 463-400, South Korea

[3]Department of Physiology, Department of Biomedical Sciences, Ajou University School of Medicine, Suwon, Korea

[4]Early Discovery, Ksilink, 16, rue d'Ankara 67000 Strasbourg, France.

Correspondence:

Kyeong Sook Choi, Professor, Department of Biochemistry, Ajou University School of Medicine, Suwon, Korea. Tel: +82-31-219-4552. E-mail: kschoi@ajou.ac.kr

Yong-Jun Kwon, Head of Early Discovery & Technology Development, Ksilink, 16, Rue d'Ankara 67000 Strasbourg, France. Tel: +33-6-3706-6662. E-mail: yjkwonipk@gmail.com.

Supplemental Materials

Chemicals and antibodies

Nonyl Acridine Orange (NAO) and Hoechst 33342 were purchased from Molecular Probes (Eugene, OR, USA). We used antibodies against p53 and Beclin-1 (Santa Cruz biotechnology, Santa Cruz, CA); p-AMPK (Cell Signaling, Beverly, MA).

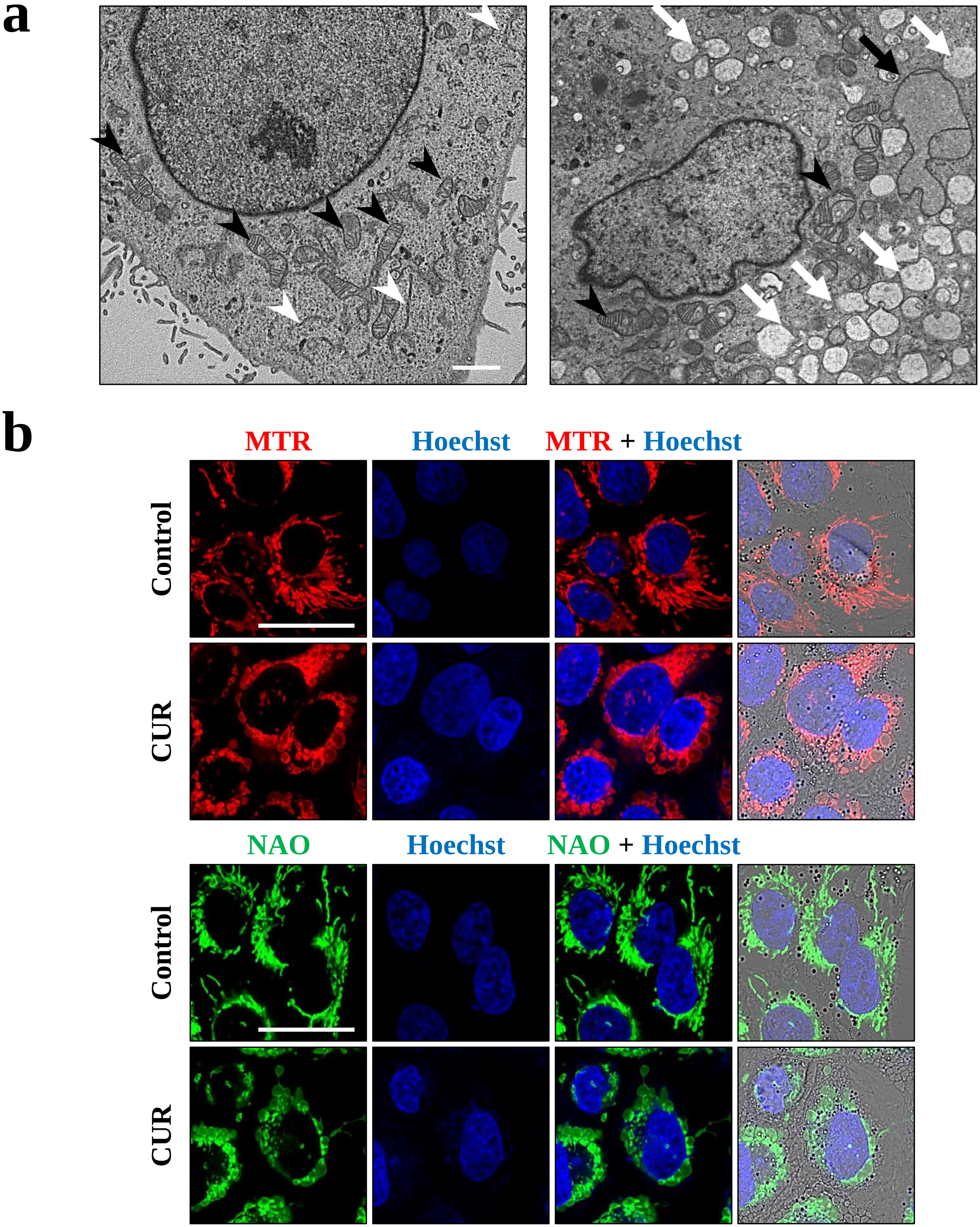

Figure S1. Curcumin induces mitochondrial dilation leading to megamitochondria formation. **(a)** MDA-MB 435S cells were left untreated or treated with 30 μM curcumin for 12 h. Transmission electron microscopy was performed. The black arrowhead and white arrowhead indicate mitochondria and the ER, respectively; the black arrow and white arrow indicate dilated mitochondria and dilated ER, respectively. Bar, 2 μm. (**b**) Cells were treated with 40 μM curcumin for 12 h, stained with 200 nM MitoTracker Red (MTR) or 200 nM Nonyl Acridine Orange (NAO) for 20 min, and observed by confocal microscopy. Bar, 30 μm.

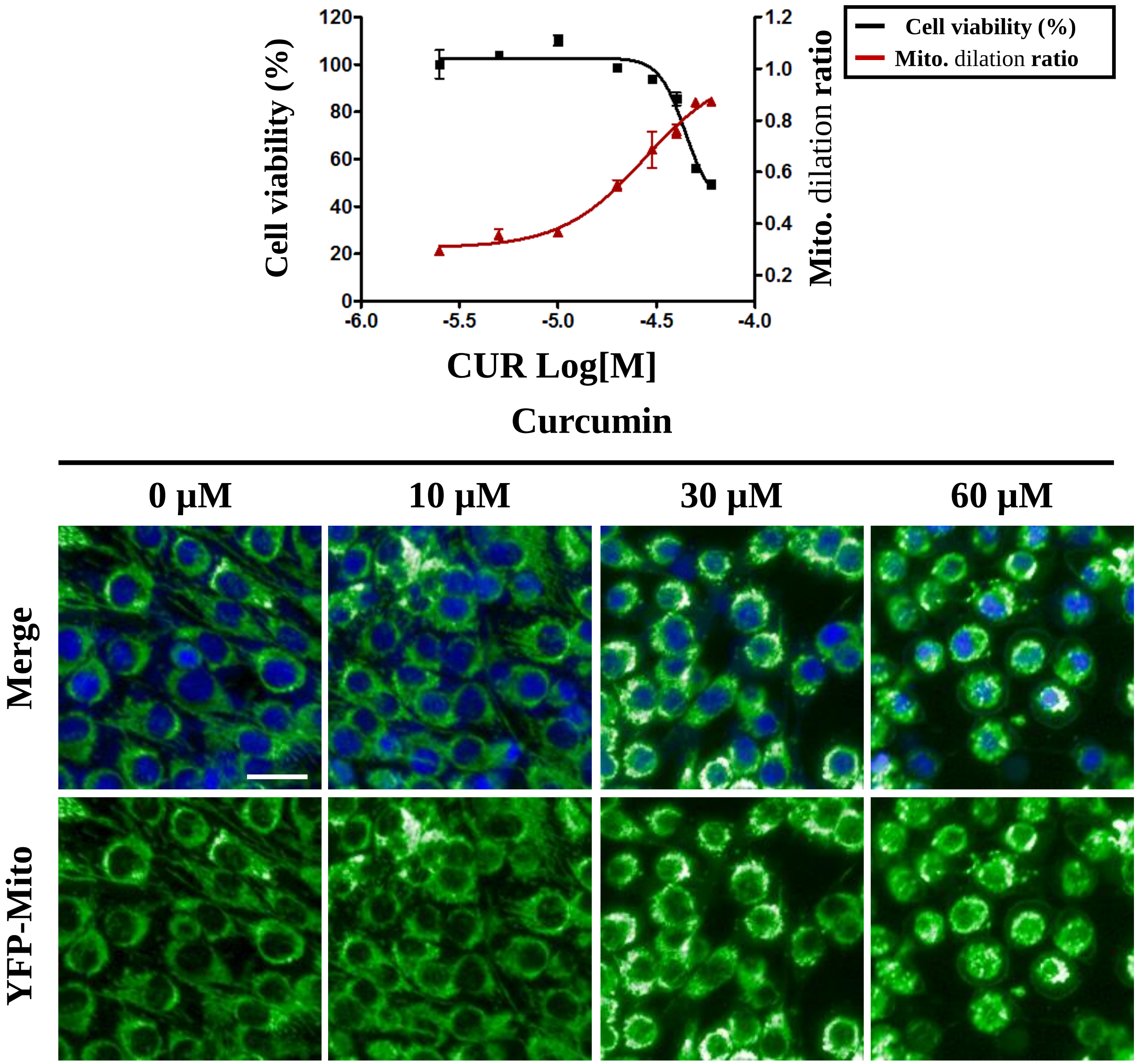

Supplementary Figure S2. Adapting the assay to a 384-well microtiter plate format. YFP-Mito cells were treated with the indicated concentrations of curcumin for 24 h. Viability and mitochondrial dilation ratios were quantitatively measured using the Columbus 2.3 software (upper panel). Cells were observed under an Opera microscope system. Bar, 50 μm (lower panel).

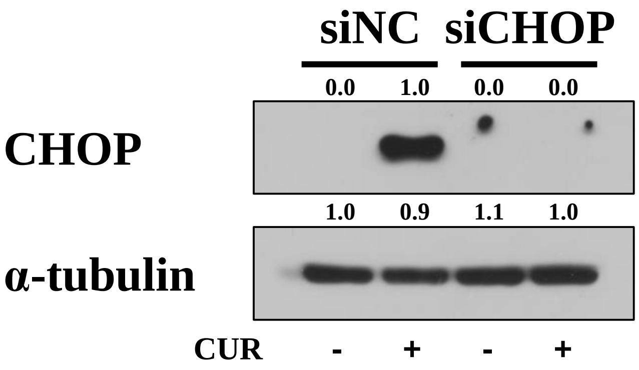

Supplementary Figure S3. Effect of siRNA-mediated CHOP knockdown. YFP-Mito cells were transfected with non-targeting siRNA or CHOP siRNA and further treated with 30 μM curcumin for 12 h. Knockdown of CHOP was confirmed by Western blotting, with α-tubulin detected as a loading control. Full-length blots are presented in Supplementary Figure S7.

Selection Hits of Curcumin-Induced Mitochondrial Dilation Assay							
Index	Name	Locus ID	zScore: Mito ratio(% Control)	Index	Name	Locus ID	zScore: Mito ratio(% Control)
1	CAMK2B	816	-2.95	20	UQCRC1	7384	-2.37
2	PRKACG	5568	-2.78	21	TNK2	10188	-2.33
3	MARK4	57787	-2.77	22	LOC51255	51255	-2.25
4	SSTR5	6755	-2.68	23	MKNK2	2872	-2.23
5	TAAR5	9038	-2.68	24	UBE2U	148581	-2.22
6	USP10	9100	-2.61	25	MYLK	4638	-2.22
7	PRKAG3	53632	-2.56	26	CTDSP2	10106	-2.20
8	PTPLB	201562	-2.55	27	LCK	3932	-2.18
9	NT5C	30833	-2.49	28	GPR15	2838	-2.16
10	INSRR	3645	-2.49	29	KUB3	91419	-2.15
11	SSTR3	6753	-2.48	30	EDG2	1902	-2.11
12	TAAR9	134860	-2.48	31	PIK4CB	5298	-2.10
13	HSPB8	26353	-2.45	32	DUSTYPK	25778	-2.09
14	PPAP2C	8612	-2.44	33	DF	1675	-2.08
15	G6PC2	57818	-2.42	34	PPP3CA	5530	-2.07
16	LOC390226	390226	-2.40	35	CCR4	1233	-2.07
17	CDK4	1019	-2.40	36	DKFZP761P0423	157285	-2.02
18	RGR	5995	-2.40	37	CDKN3	1033	-2.00
19	GPR56	9289	-2.39	38	GPR153	387509	-2.00

Supplementary Table S1. Selected hits for genes that appear to be involved in curcumin-induced mitochondrial dilation. Lists of selected hits with Z-scores < -2.

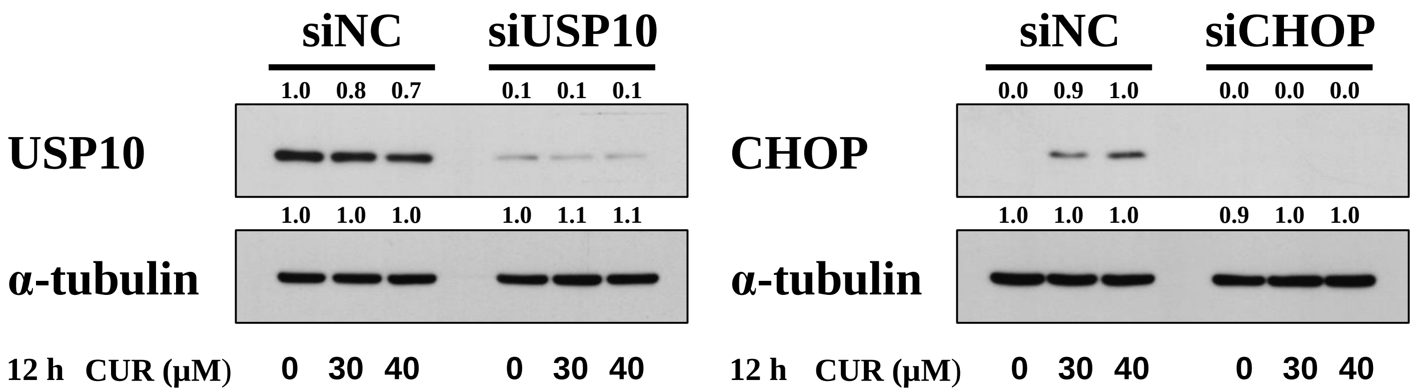

Supplementary Figure S4. Effect of siRNA-mediated USP10 and CHOP knockdown. YFP-Mito cells were transfected with non-targeting siRNA or CHOP siRNA and further treated with the indicated concentrations of curcumin for 12 h. Knockdown of USP10 or CHOP were confirmed by Western blotting, with α-tubulin detected as a loading control. Full-length blots are presented in Supplementary Figure S7.

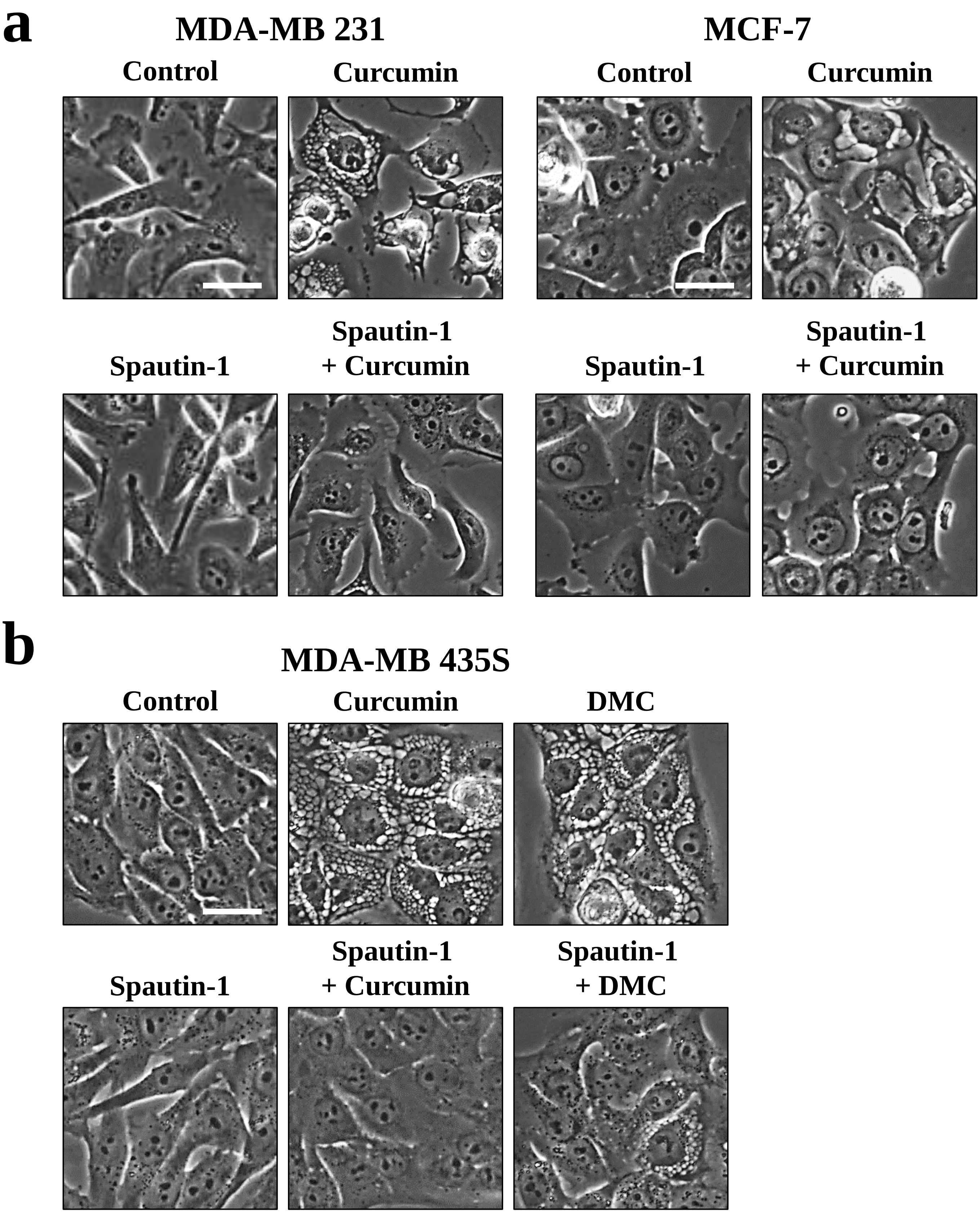

Supplementary Figure S5. Spautin-1 effectively inhibits curcumin-induced cytoplasmic vacuolation in various cancer cells. **(a)** MDA-MB 231 or MCF-7 cells pretreated with or without 5 μM spautin-1 were further treated with 40 μM curcumin for 12 h and observed by phase-contrast microscopy. Bar, 10 μm. **(b)** MDA-MB 435S cells pretreated with or without 5 μM spautin-1 were further treated with 40 μM curcumin or 20 μM DMC for 12 h and observed by phase-contrast microscopy. Bar, 10 μm.

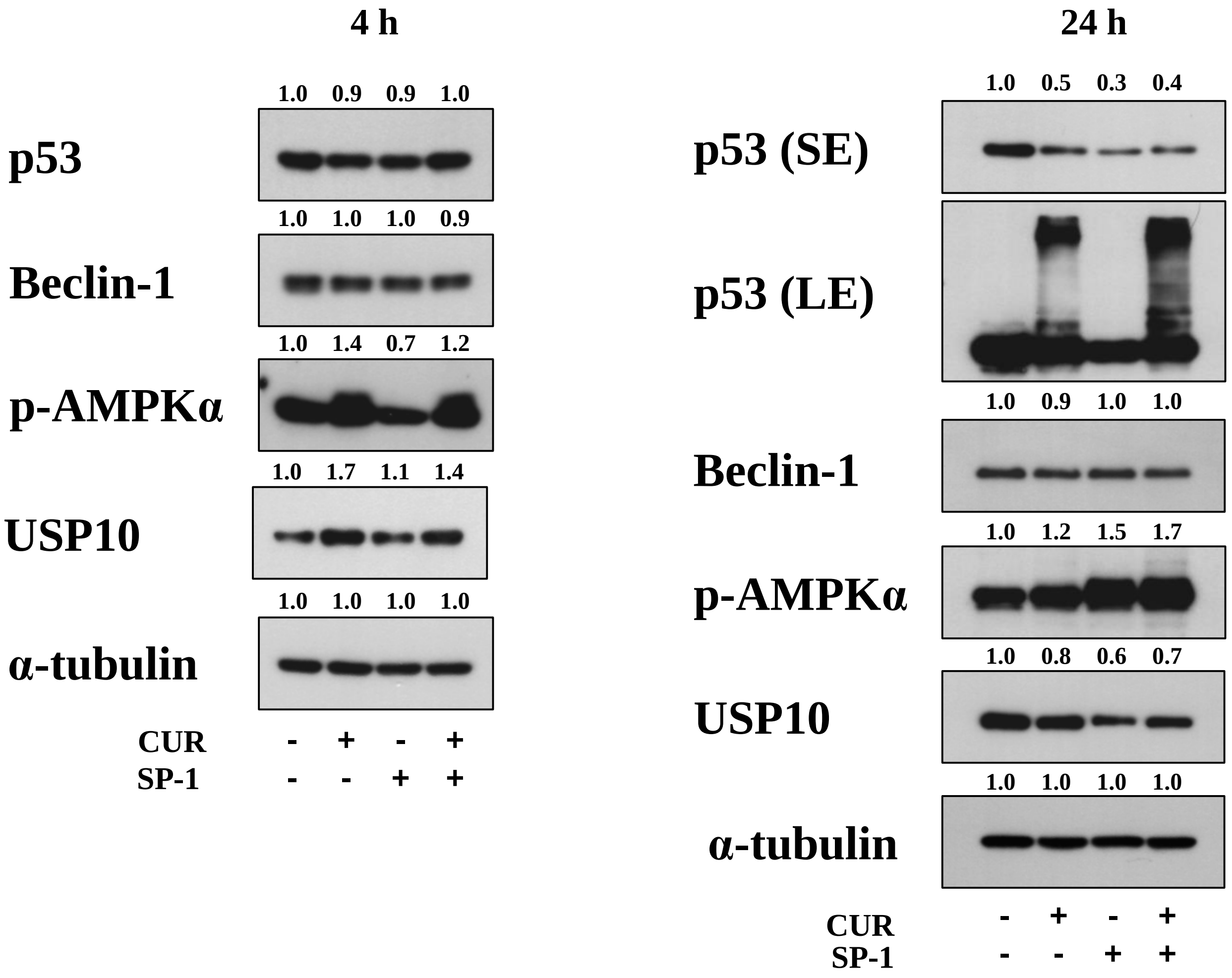

Supplementary Figure S6. MDA-MB 435S cells treated with 40 μM curcumin and/or 10 μM spautin-1 for the indicated time points were subjected to Western blotting of the indicated proteins, with α-tubulin detected as a loading control. Full-length blots are presented in Supplementary Figure S7.

Fig. 6a

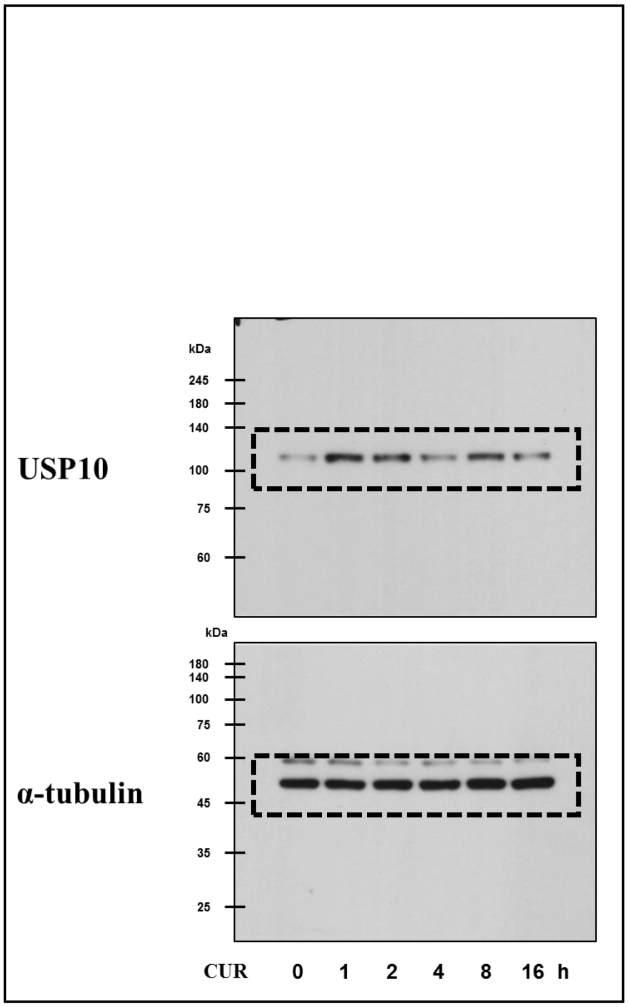

Fig. 6b

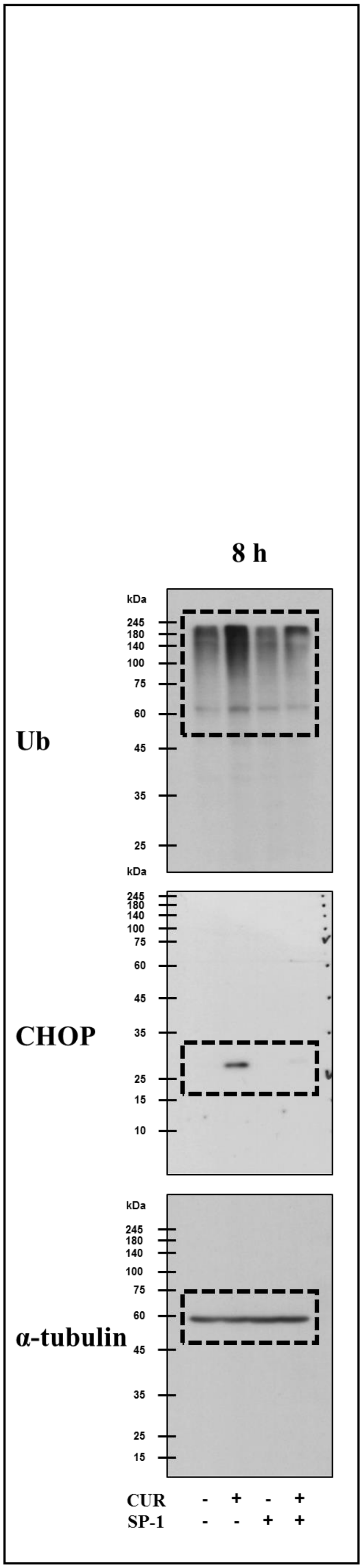

Fig. 6c

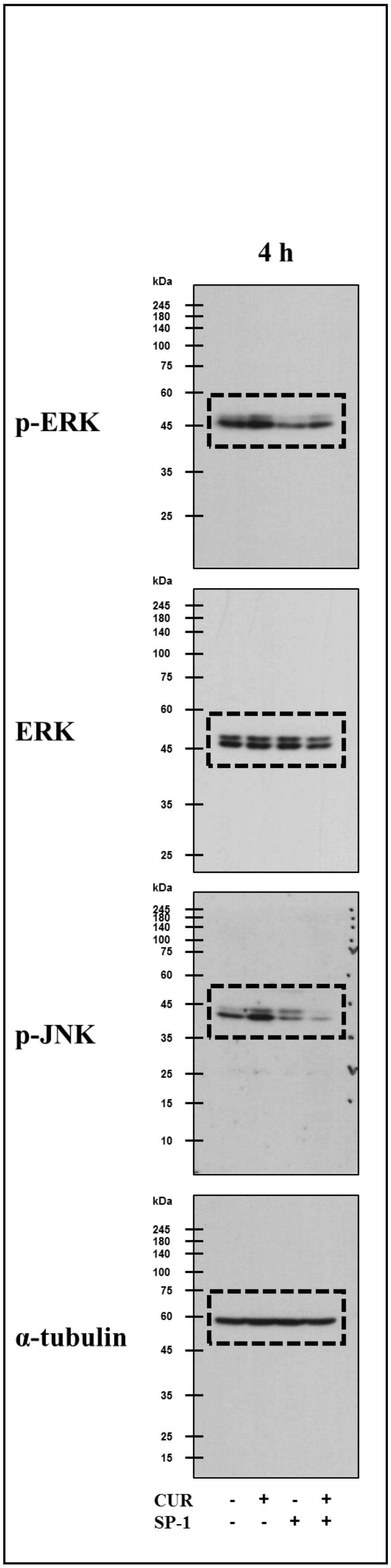

Supplementary Fig. S3

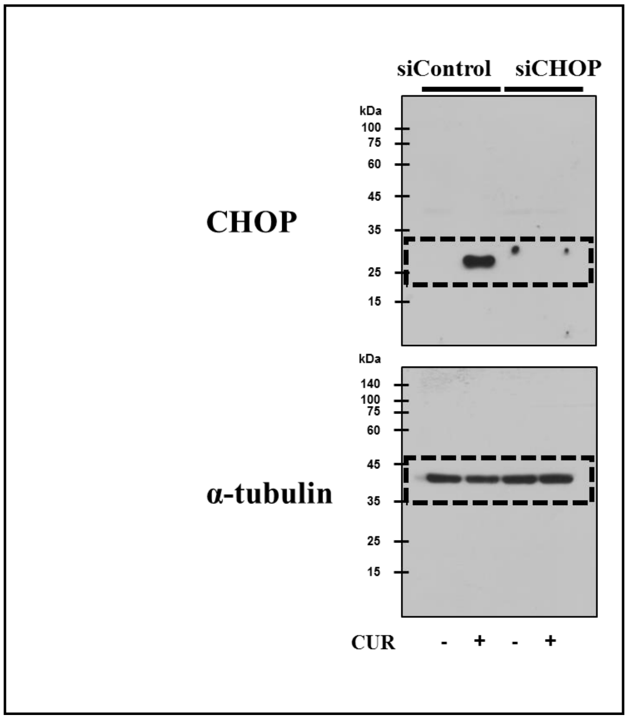

Supplementary Figure S7. Full-length images of western. The black dotted lines on the western blotting indicate the cut outlines of the cropped images used in Figure 6, Supplementary Fig. S3, Supplementary Fig. S4. and Supplementary Fig. S6.

(Continued)

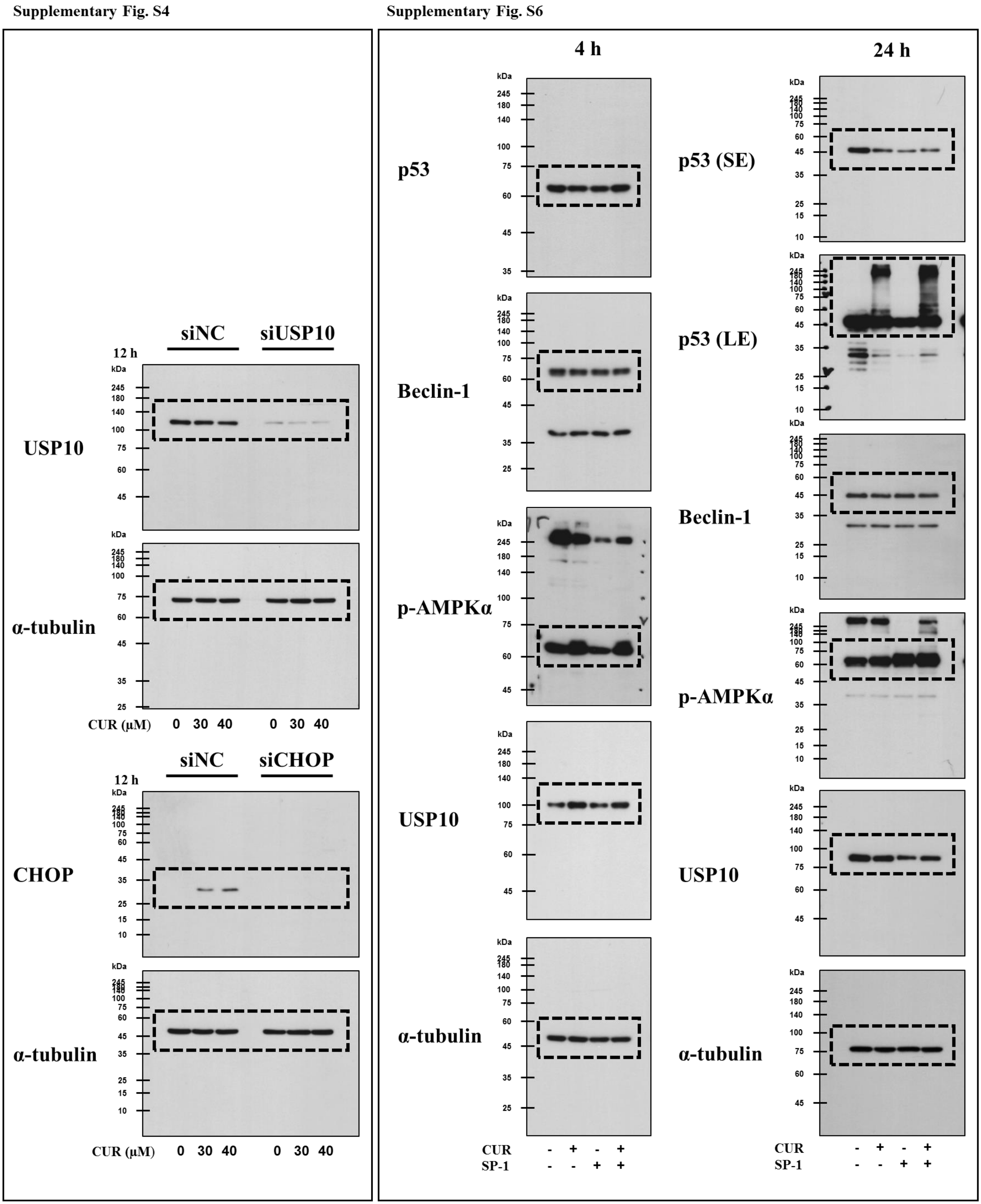

Supplementary Figure S7. Full-length images of western. The black dotted lines on the western blotting indicate the cut outlines of the cropped images used in Figure 6, Supplementary Fig. S3, Supplementary Fig. S4. and Supplementary Fig. S6.
